# A Systematic Review and Meta-Analysis of the Efficacy of Biosecurity in Disease Prevention and Control in Livestock Farms in Africa

**DOI:** 10.1155/2024/8683715

**Published:** 2024-11-14

**Authors:** Ronald Vougat Ngom, Gaspard J. Ayissi, Adonis M. M. Akoussa, Andrea Laconi, Saleh M. Jajere, Henriette A. Zangue, Alessandra Piccirillo

**Affiliations:** ^1^School of Veterinary Medicine and Sciences, University of Ngaoundere, Ngaoundere 454, Cameroon; ^2^Department of Comparative Biomedicine and Food Science, University of Padua, Legnaro 35020, Italy; ^3^Department of Comparative Biomedicine and Food Science, University of Padua, Viale dell'Università 16, Legnaro 35020, Italy; ^4^National School of Agro-Industrial Sciences, University of Ngaoundere, Ngaoundere 454, Cameroon

**Keywords:** Africa, African swine fever, avian influenza, biosecurity, cattle, coccidiosis, pigs, poultry, review

## Abstract

In Africa, livestock production plays a crucial role for sustainable food security and economic growth. However, the development of this sector has been delayed by livestock diseases, one of the main constraints, which can cause important production and economic losses. To overcome these constraints, farmers extensively use antimicrobials, which in turn can lead to antimicrobial resistance (AMR), one of the main threats to global health and food security. Biosecurity has been identified as a key strategy to reduce livestock diseases. Therefore, the current systematic review and meta-analysis, conducted according to the Cochrane guideline, aimed at determining the efficacy of biosecurity in preventing and controlling infectious diseases in livestock farms in Africa. Of the 1408 records retrieved from five different databases, only 16 met the inclusion criteria. These studies were conducted in Egypt (31.2%), Nigeria (31.2%), Uganda (18.8%), Ethiopia (12.5%) and Tunisia (6.3%) and concerned poultry (62.4%), pigs (18.8%) and cattle (18.8%). Investigations focused mainly on avian influenza (AI) (15.0%) and coccidiosis (10.0%) in poultry and African swine fever (ASF) (10.0%) in pigs. In poultry farms, the results of the pairwise meta-analysis showed that biosecurity measures related to visitors and farmworkers could be effective at reducing the risk of introduction and spread of AI viruses (odds ratio [OR] = 0.48; 95% confidence interval [CI] 0.28–0.82). Moreover, inadequate biosecurity seemed to be a factor promoting coccidiosis (OR = 4.20; 95% CI 2.4–7.4) and AI (OR = 1.74; 95% CI 1.23–2.48). Prevention of ASF was significantly associated with the application of biosecurity measures related to animals' transport, removal of carcasses and manure (OR = 0.33; 95% CI 0.12–0.88). Despite their importance, these findings cannot be translated to the entire African continent, since no studies were available for more than 90% of its countries. More research should be carried out to fill in the gaps identified by this review.

## 1. Introduction

Population growth and increasing disposable incomes are among the main drivers influencing food-producing animal industry in Africa [1, 2], and livestock production plays a crucial role in most African countries. It significantly contributes to food security, livelihoods improvement, economic growth and sustainable development [2]. About 50% of African household food requirements and income depend directly or indirectly on livestock farming [3]. However, the livestock sector faces a number of challenges such as infectious diseases, which are crucial constraints due to their impacts in terms of morbidity and mortality, production losses and costs associated with disease management [4, 5]. These constraints are linked to poor husbandry management, high human–animal–environmental interactions and increased intensification of smallholder livestock farms. Indeed, smallholder farmers represent the main stakeholders of the livestock sector in Africa [6].

In several African countries, efforts to eradicate livestock diseases have been limited by technical, financial, logistical [7] or socioeconomic constraints. Therefore, to prevent and control infectious diseases, the majority of African livestock farmers extensively uses antimicrobials [8, 9]. In addition, antimicrobials are used as growth promoters to boost farms' productivity [10]. The extensive and imprudent use of antimicrobials in livestock in Africa can lead to the presence of antimicrobial residues in foods of animal origin [11, 12]. In addition, antimicrobial use (AMU) has been associated with the emergence and spread of antimicrobial resistance (AMR) in animals, humans and the environment [8, 13]. Widely referred as the ‘silent pandemic', AMR is a global health concern with the highest burden reported in Africa [14], probably due to weak health systems and limited resources. A recent comprehensive study covering data from 47 of the 54 African countries and inclusive of 23 bacterial pathogens and 88 pathogen–drug combinations revealed an alarming finding: an estimated 1.05 million deaths were associated with bacterial AMR, and 250,000 deaths were directly attributed to bacterial AMR in 2019 [15].

In this context, the development of new strategies to prevent infectious diseases in livestock farms and therefore reduce antimicrobial usage is of utmost importance. Preventive livestock healthcare is a holistic approach aiming at reducing health threats and promoting more efficient and productive livestock. Many studies presented biosecurity as a key strategy that can contribute to reduce disease transmission and AMU [16, 17]. Indeed, biosecurity is defined as a set of management and physical measures designed to reduce the risk of introduction, establishment and spread of animal diseases, infections or infestations to, from and within an animal population [18]. Biosecurity measures help either in reducing the risk of entry of new pathogens (external biosecurity) or on reducing their spread within the farm (internal biosecurity). External biosecurity measures are barriers or rules banning the introduction of certain animals, people or vehicles that could introduce pathogens in the farms, while internal biosecurity measures are related to the management of the herd, general hygiene of facilities and cleaning and disinfection and farmworkers [16–18]. Therefore, this systematic review and meta-analysis was conducted with the aim to determine the efficacy of biosecurity measure implementation for controlling infectious diseases in livestock farms in Africa.

## 2. Materials and Methods

The Preferred Reporting Items for Systematic Reviews and Meta-Analyses (PRISMA) standards [19] were followed for reporting this study.

### 2.1. Protocol and Registration

As recommended, the protocol of this review was first developed and registered in the Systematic Reviews for Animals and Food (SYREAF) website [20].

### 2.2. Eligibility Criteria

The criteria used for eligibility for this review were based on the Population (cattle, pigs, poultry, sheep or goats in farms across African countries), Intervention (biosecurity measures), Comparator (no biosecurity) and Outcome (disease prevalence/occurrence) (PICO) framework. Poultry species were limited to broilers, layers, ducks, turkeys and geese. The animal species included in this review were selected due to their relevance in the African farming system in terms of livestock production [13, 20]. Considering the language of the authors, studies in English or French were included, and no restrictions were imposed on the publication date of the studies.

### 2.3. Sources of Information

A comprehensive literature search was performed as described previously [21]. Briefly, CAB Abstracts (Ovid interface), Agricola (in ProQuest), Web of Science (WOS), Scopus and PubMed databases via the University of Bern (Switzerland) were used for the search. The search in all the selected databases was performed in August 2023. The following databases were excluded in WOS during the search: Arts & Humanities Citation Index (A&HCI), Conference Proceedings Citation Index-Science (CPCI-S) and Conference Proceedings Citation Index-Social Science & Humanities (CPCI-SSH).

### 2.4. Search Strategy

The search strategy included each part of our PICO framework, except for the disease part that was not used for two reasons: (i) the high number of animal diseases and (ii) the limited number of studies that assessed biosecurity in livestock farms in Africa [21]. Finally, the same search strategy used in our previous review was applied as follows: [biosecurity] AND [cattle or poultry or pigs or sheep or goats] AND [farm] AND [African countries].

### 2.5. Study Selection

Records were downloaded from databases, and duplicates were removed using Zotero (version 6.0.30). The obtained files were uploaded in Rayyan (https://www.rayyan.ai/) for the screening process. All retrieved items underwent an initial screening based on title and abstract to assess their relevance to the subject. If considered pertinent to the present review, corresponding full-text versions were downloaded (using PubMed via the University of Padua library) and screened. For both screening phases, two pair of reviewers screened bibliographic records independently. For each pair of reviewers, disagreements were either resolved by consensus or, when consensus was not reached, through input from a third reviewer. Before each screening phase, a calibration exercise was performed among all the reviewers to maintain consistency, as recommended by Paudel et al. [22]. First and second pretest were performed on 50 and 10 randomly selected records, respectively. During calibration, an agreement of at least 90% was obtained among all the reviewers before starting the screening.

For the first phase of the screening, the eligibility of studies was assessed using the following set of questions:

1. Does the study concern at least one of the following species: poultry, cattle, pigs, sheep and goats? Yes (include), no (exclude) and unclear (include)
2. Is the study original research? Yes (include), no (exclude) and unclear (include)
3. Does the study take place in at least one African country? Yes (include), no (exclude) and unclear (include)
4. Does the study concern biosecurity assessment? Yes (include), no (exclude) and unclear (include)
5. Does the study concern a livestock disease or pathogen in farms? Yes (include), no (exclude) and unclear (include)

The full-text screening included the following questions:

1. Is a full text available? Yes (include) and no (exclude)
2. Is the full text available in English or French? Yes (include) and no (exclude)
3. Does the study concern biosecurity assessment at farm/no risk factor study? Yes (include) and no (exclude)
4. Does the study provide evidence on the presence of diseases/pathogens? Yes (include) and no (exclude)
5. Has the association between biosecurity measures and the diseases/pathogens been assessed? Yes (include) and no (exclude)

### 2.6. Data Extraction

A data extraction Microsoft Excel (version 2020) spreadsheet was developed by the first author and validated by all the authors after a pretest performed by extracting data from eight randomly selected papers [13]. Three independent reviewers performed the data extraction from the included papers. Data from each paper were extracted by two persons and then checked by a third one. When needed, disagreements were resolved between the three reviewers. Data extracted from eligible studies included first author, year of publication, period of data collection, country, species (cattle, poultry, pigs, sheep and goats), sample size (number of farms/animals), disease/pathogen studied, number of positive samples and list of biosecurity measures studied for their association with the disease or the pathogen. The Excel sheet containing all the variables is available upon request to the first author.

### 2.7. Quality Assessment of the Selected Studies

The assessment of the quality of the studies was performed as described by Yang et al. [23], by using the Grading of Recommendations Assessment, Development and Evaluation (GRADE) system [24]. The authors independently assessed the risk of bias of five domains based on the following questions:

1. Domain 1: Sampling time was definite?
2. Domain 2: Sampling method was described in detail?
3. Domain 3: Samples were collected randomly?
4. Domain 4: Detection method was clarified?
5. Domain 5: Four or more factors (i.e. biosecurity measures) were studied?

If the answer of a domain was ‘yes', one point was awarded; otherwise, ‘zero' was given. Thus, each paper was classified in one of the following categories: high-quality (score = 4–5 points), average-quality (score = 2–3 points) and low-quality articles (score = 0–1 point).

### 2.8. Data Synthesis and Statistical Analysis

**Figure 1:**
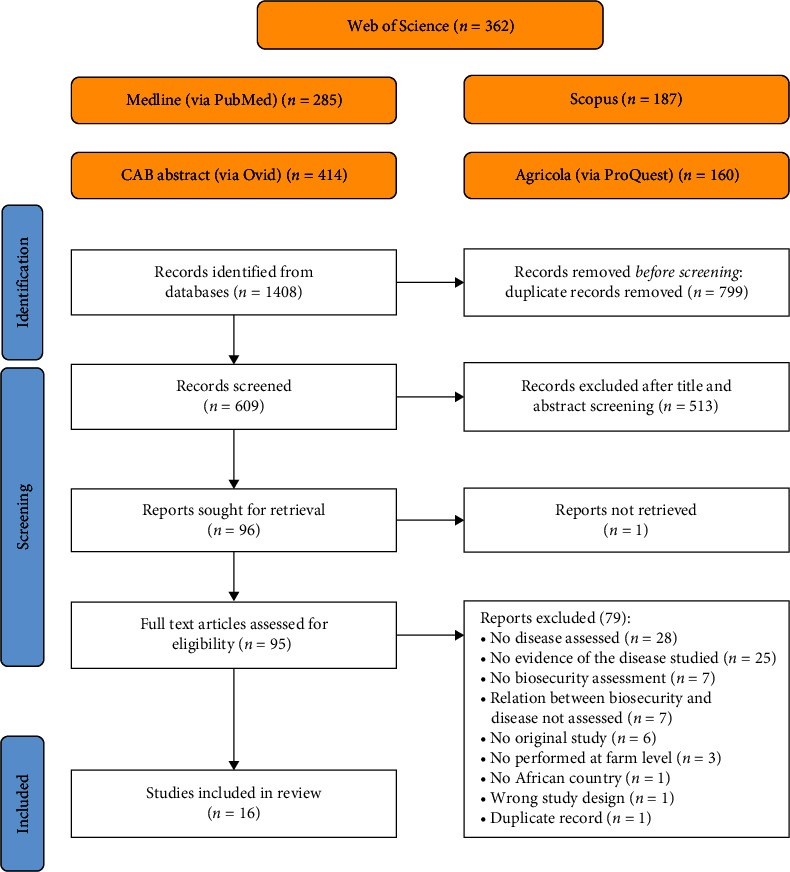
Flow diagram illustrating the selection of eligible studies. This flow diagram presents the selection process and the number of papers selected in each step.

The results of the literature search and the records selection are provided in a flow diagram (Figure 1). Descriptive statistics of the characteristics of the included studies were narratively summarized after tabulation using Microsoft Excel 2020. The pairwise meta-analysis was performed using Revman (version 5.4.1.), as described by Wang et al. [25] with slight modifications. To avoid misclassification caused by unclear definitions of the factors evaluated, for each animal species (i.e broiler, layer and pig), biosecurity measures were classified in groups (i.e., removal of manure and carcasses, cleaning and disinfection and materials and measures between compartments) according to Biocheck.UGent (https://biocheckgent.com/en/). For a specific disease of the same animal species, meta-analysis was performed only when data on at least three measures within a group were available. The merged effect was the odds ratio (OR) and its 95% confidence interval (CI), which reflected the strength of the association between biosecurity measure and disease. The Cochran's test based on chi-square was used to assess heterogeneity, and a significance level of 5% was set. Random effects model was used to estimate the overall OR of the studies [26]. Heterogeneity was considered high when the *I*^2^ ≥ 75%, moderate when *I*^2^ >50% and low when *I*^2^ ≤50%. The *p*-value was set at 0.05. A group of measure is a protective factor for a disease when the OR <1, *I*^2^ ≤ 50% and *p* ≤ 0.05. Forest plot for one meta-analysis is included to show the methodology employed for the analyses.

### 2.9. Risk of Bias Across Studies

Publication bias was assessed by using funnel plot and Egger's weighted regression tests [27, 28]. A *p*-value <0.05 was considered statistically significant for all tests.

## 3. Results

### 3.1. Study Selection

The search in the five databases resulted in 1408 records, of which 799 were excluded following deduplication. After title and abstract screening, 513 records were also removed. Out of 96 records assessed during full-text screening, only 16 papers were included in this review. The majority of the records were excluded since they did not assess diseases or pathogens (*n* = 28) or they did not provide evidence of the presence of the disease (*n* = 25) (Figure 1).

### 3.2. Characteristic and Quality of Included Studies

The characteristics of the included studies are reported in Table 1. All selected studies (*n* = 16) were carried out across five African countries (i.e. Egypt, Ethiopia, Nigeria, Tunisia and Uganda) and published in English after 2011 (Table 1). The majority was conducted in Egypt (*n* = 5) and Nigeria (*n* = 5) and published between 2020 and 2023 (*n* = 7). Most of the studies were cross-sectional (*n* = 12) and performed in poultry farms (*n* = 10). When considering animal diseases, three studies focused on avian influenza (AI) and two on African swine fever (ASF). In addition, as presented in Table 2, quality assessment of the selected records showed that six studies were of high quality (4 or 5 points), eight of average quality (2 or 3 points) and two of low quality (0 or 1 point).

### 3.3. Biosecurity Measures Associated With Livestock Diseases in Africa

Summary of the biosecurity measures and diseases/pathogens assessed in each study is reported in Table 3. Out of the 16 studies included, six focused on one disease (i.e. AI, ASF, bovine tuberculosis and lumpy skin disease). Four studies assessed the presence of *Salmonella* spp. alone (*n* = 2) or in combination with other bacteria (*n* = 2). Others evaluated the prevalence of pathogens such as *Escherichia coli* (*n* = 1), *Staphylococcus* spp. (*n* = 1) and thermophilic *Campylobacter* spp. (*n* = 2).

In six studies, the association between disease and biosecurity measures was evaluated (i.e. prevalence of AI in farms where the ‘all-in/all-out' system was not applied), but the dependence of the relation was not assessed. In others (*n* = 6), the strength of the association between biosecurity measures and the diseases/pathogens was quantified. Therefore, data for the meta-analysis on the biosecurity factors associated with livestock diseases in the African context were extracted from these late six studies [1, 10, 19, 30, 33, 35]. Among them, four focused on one disease and the others on at least two diseases/pathogens. The diseases and associated biosecurity measures reported in the six studies are presented in Table 4. In the selected studies, few internal biosecurity measures were considered as risk factors.

### 3.4. Synthesis of Results

The meta-analysis results showed that ASF and AI were significantly associated with factors related to the transport of animals, removal of carcasses and manure (*p*  < 0.0001) and measures concerning visitors and farmworkers (*p*  < 0.007), respectively. Both groups of measures would have been protective factors (OR <1). However, in the case of AI, high heterogeneity (*p*  < 0.00001, *I*^2^ = 96%) among the included studies or biosecurity measures was identified. In the case of ASF, measures related to visitors and farmworkers (0.74 [0.49, 1.13]) and cleaning and disinfection (0.79 [0.50, 1.24]) recorded an OR <1, but their 95% CI included 1 which means that they could not be considered as protective factors.

**Figure 2:**
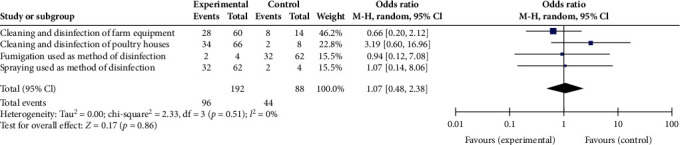
Forest plot of the efficacy of biosecurity measures related to cleaning and disinfection to control coccidiosis in poultry in Africa. CI, confidence interval.

Only poor implementation of biosecurity measures was recorded as a possible risk factor for AI (1.74 [1.23, 2.48], *p* < 0.002) and coccidiosis (4.20 [2.4, 7.4], *p* < 0.001) in poultry. Three additional groups of possible risk factors associated with diseases, although not statistically significant (*p* > 0.05), were vermin and birds control for ASF (OR = 1.55), infrastructure and biological vectors (OR = 1.49) for AI and cleaning and disinfection (OR = 1.07) for coccidiosis (Figure 2). Many groups of biosecurity measures showed no association with any disease (Table 5 and Figure 2).

A high heterogeneity (*I*^2^ >75%) was recorded among the studies for the majority of the biosecurity measure groups. Thus, concerning ASF, biosecurity measures related to vermin and bird control (*I*^2^ = 96%), cleaning and disinfection (*I*^2^ = 96%) and measures between compartments, working lines and use of equipment (*I*^2^ = 95%) showed a high heterogeneity. Similar result was found for biosecurity measures in relation with visitors and farmworkers (*I*^2^ = 97%) and infrastructure and biological vectors (*I*^2^ = 96%) when associated with AI. Only measures related to transport of animals, removal of carcasses and manure for controlling ASF and all the measures for controlling coccidiosis showed a nonsignificant high homogeneity (*I*^2^ = 0%–50%). Due to the limited number of studies included in the meta-analysis and the low number of factors within each group of biosecurity measures, sensibility analysis was not performed.

## 4. Discussion

This systematic review and meta-analysis summarizes biosecurity measures associated with disease prevention and control in livestock (cattle, pigs and poultry) farms in Africa. Only 16 studies meeting the selection criteria were included in this review. All studies were conducted in only five African countries and published after 2011. These findings highlight the need for more research in this area to improve the understanding of the epidemiology of infectious diseases in livestock and their prevention and control measures in the African context. The majority of studies investigated AI and coccidiosis in poultry and ASF in pigs. Poultry and pigs represent the main food-producing animal species in Africa. A recent study reported that by 2050, the projected demand for poultry and pig meat in some sub-Saharan countries would increase by 214% and 161%, respectively [45]. The aforementioned diseases are known to have the highest impact and burden on poultry and pig productions, not only in Africa, but also worldwide.

Meta-analysis revealed that biosecurity measures related to visitors and farmworkers were effective at minimizing the risk of introduction of AI (OR <1). These bioexclusion procedures included vehicle restriction at farm gates, access control and cleaning and disinfection of people entering the farm (changing shoes, washing hands etc.). This result agrees with findings reported in previous studies. For instance, Patyk et al. [46] found that workers showering before entering the farm was a protective factor for the introduction of AI in commercial turkey farms. Similarly, prohibiting the access of vehicles to the poultry sheds was shown to reduce the circulation of AI virus in commercial chicken farms in Bangladesh [47]. Green et al. [48] reported that the presence of a gate at the farm entrance and changing clothes for workers entering the poultry barns is a protective factor. Our findings suggest that inadequate implementation of biosecurity measures could also be considered an important risk factor (OR >1) for AI. This finding should not be a surprise since biosecurity represents the main strategy for disease prevention and control in poultry production. However, in Africa, little efforts have been made in the poultry production chain to ensure the implementation of appropriate biosecurity measures. This may be linked to limited resources available to African poultry farmers. Indeed, to be effective, biosecurity measures should be implemented continuously. Furthermore, the lack of regulations on biosecurity and/or their enforcement in livestock farming in Africa may contribute to the low level of biosecurity compliance in farms [21]. AI is a notifiable disease [49] that can cause huge economic losses in the poultry industry and may pose a serious threat to public health [50, 51]. Therefore, the identification of risk and protective factors for AI is crucial for the African poultry sector due to the high prevalence (8.0%, 95% CI 2.2–25.7) recorded in the continent [23].

Poor management practices showed to be a possible significant risk factor for coccidiosis in poultry farms in Africa. This is not surprising because factors, such as poor hygiene of personnel, poor cleaning of equipment and presence of rodents and insects, can increase the spread of this disease [52], highlighting once more the importance of biosecurity for disease control. Accordingly, Safae et al. [53] reported higher coccidiosis infection rates in farms showing inadequate biosecurity implementation in comparison to those with good level of biosecurity practice. Commonly, coccidiosis is tackled by using coccidiostats in poultry feed or by vaccination [54, 55]; however, the presence of residues of coccidiostats in poultry meat products is of concern, since they may pose a risk to public health [56, 57]. In recent years, several studies have reported the increasing resistance to anticoccidials as a major constraint and limiting factor for commercial poultry farmers, which can result in the capital/resources wastage on coccidiosis control [58–60]. This seems to suggest that proper biosecurity measure implementation remains the best option for the prevention and control of coccidiosis especially in Africa where a pooled prevalence of coccidiosis as high as 39.9% has been reported [61]. Coccidiosis, as one of the most significant parasitic diseases of poultry, costs the world's commercial chicken producers at least US$ 1.5 billion per year [62]. Indeed, infections with *Eimeria* spp. cause poor performances both in terms of feed conversion and productivity in poultry [63]. A recent estimation of the associated costs of coccidiosis in poultry from six continents, including Africa, indicated an estimated global cost of £10.4 billion (£7.7–£13.0 billion) equivalent to £0.16/chicken produced [64].

ASF, a highly contagious viral disease of pigs with no effective vaccine available, is currently the most serious disease affecting the pig industry worldwide, characterized by a nearly 100% mortality rate [65, 66]. Its effects are severe in smallholder and traditional farming systems with inappropriate biosecurity practice. An estimated loss of over US$ 2.9 billion per year has been reported by Fadiga, Christine and Ihedioha [67] in sub-Saharan Africa alone. In the current review, many biosecurity measures have been studied, and their association with ASF has been explored. However, only implementation of biosecurity measures related to the transport of animals, removal of carcasses and manure has been found to be significantly associated with a reduction of ASF. These biosecurity measures included routinely removal of manure and litter, prompt disposal of dead animals and safe disposal of faeces and carcasses in African piggeries. Indeed, these actions can significantly contribute to reduce the dissemination of ASF virus (ASFV) within and between farms, since, due to the uniqueness of the biological properties and long-term preservation in the environment of ASFV, carcasses and faeces represent important sources for the horizontal transmission of the virus [68–70]. Similar practices have been previously recommended as preventive measures for limiting the spread of ASF [71, 72]. Since the African pig population has seen an exponential growth in recent years, coupled with the endemicity of ASF in many countries, as well as its negative contribution to serious economic losses, the implementation of the aforementioned biosecurity measures could significantly contribute to the reduction of ASF in Africa [73, 74].

Finally, as transboundary animal diseases, building strong collaborations at regional, national and international level is crucial for the control and prevention of ASF and AI in Africa. The same approach should be considered for coccidiosis. This includes establishing more stringent monitoring and surveillance programmes as well as biosecurity legislation. At country level, effective prevention and control of livestock diseases require inputs from all the stakeholders involved in the animal food production systems, including national authorities, farmers and other key actors (e.g. epidemiologists and socioeconomists). In Africa, national animal health authorities should reinforce/revise/update the development and implementation of appropriate policies/regulations/programmes for the prevention and control of livestock diseases. Furthermore, achieving sustainable intensification of the farming system will require implementing strict biosecurity measures and call for increased investment by livestock farmers.

## 5. Limitations

The results of this systematic and meta-analysis should be interpreted with caution. First, not only few studies were included, but they also showed high heterogeneity. No study from central and South Africa countries was found. These might affect the accuracy of the conclusions, and the generalizability of these results to the whole continent. In addition, this review focused on peer-reviewed articles; thus, grey literature and unpublished studies were not included. Finally, in some papers, biosecurity measures were not clearly defined. This could have led to misclassification of these measures.

## 6. Conclusion

This systematic review and meta-analysis were conducted to determine the efficacy of biosecurity measures for controlling infectious diseases in livestock farms in Africa. The review identified a limited number of studies, conducted in only five African countries. However, the meta-analysis showed that biosecurity measures related to the transport of animals, removal of carcasses and manure and visitors and farmworkers could be effective in reducing the risk of introduction of ASF and AI in pig and poultry farms, respectively. Inappropriate implementation of biosecurity measures was found to be a possible risk factor for coccidiosis and AI infections in poultry farms. These findings provide new insights regarding promising targets for the intervention by the relevant authorities. Unfortunately, these results cannot be generalized to the whole African continent because of the lack of studies in more than 90% of African countries. Future research should aim at filling the gaps revealed in this review, especially focusing on those countries where such studies have never been carried out and other diseases that severely affect the African livestock production.

## Figures and Tables

**Table 1:** General characteristics of the studies included in this review.

Variables	Number of studies	Percentage	Reference
Language	16	—	—
English	16	100	[29–44]
French	0	0	—
Year of publication	16	—	—
2012–2015	4	25.0	[29, 38, 41, 42]
2016–2019	5	31.2	[30, 37, 39, 40, 44]
2020–2023	7	43.8	[30, 32–36, 43]
Country	16	—	—
Egypt	5	31.2	[29, 32, 33, 35, 36]
Ethiopia	2	12.5	[39, 40]
Nigeria	5	31.2	[37, 38, 42–44]
Tunisia	1	6.3	[41]
Uganda	3	18.8	[29, 31, 34]
Study design	16	—	—
Cross-sectional	12	75.0	[30–34, 36, 37, 39–41, 43, 44]
Cross-sectional and retrospective	2	12.5	[35, 38]
Longitudinal	2	12.5	[29, 42]
Sampling strategy for farm selection	16	—	—
Random	6	37.5	[29, 31, 32, 34, 43, 44]
Convenience	4	25.0	[38–41]
Not reported	6	37.5	[30, 33, 35–37, 42]
Animal species	16	—	—
Cattle	3	18.8	[31, 35, 39]
Pigs	3	18.8	[29, 34, 42]
Poultry	10	62.4	[30, 32, 33, 36–38, 40, 41, 43, 44]
Disease	16	—	—
Avian influenza	3	18.8	[30, 38, 41]
African swine fever	2	12.5	[29, 42]
Coccidiosis	2	12.5	[33, 40]
Bovine tuberculosis	1	6.3	[39]
Brucellosis	1	6.3	[31]
Lumpy skin disease	1	6.3	[35]
Bovine viral diarrhoea	1	6.3	[29]
Others (salmonellosis, gastrointestinal parasites, campylobacteriosis, colibacillosis)	9	56.3	[30, 32, 33, 34, 36, 37, 41, 43, 44]

**Table 2:** Quality scores of the studies included in this review.

Reference	Random sampling	Detection method	Sampling method	Sampling time	Four or more risk factors	Score	Study quality
Muhangi et al. [29]	Yes	Yes	Yes	Yes	Yes	5	High
Adel et al. [30]	No	Yes	No	Yes	No	2	Average
Wolff et al. [31]	Yes	NA	Yes	Yes	Yes	4–5	High
Solima and Abdallah [32]	Yes	Yes	No	Yes	Yes	4	High
Abouelenien et al. [33]	No	Yes	No	Yes	Yes	3	Average
Oba et al. [34]	Yes	Yes	Yes	Yes	No	4	High
Ezzeldin et al. [35]	No	NA	No	Yes	Yes	2–3	Average
Abouelenien et al. [36]	No	No	No	Yes	Yes	2	Average
Ola-Fadunsin et al. [37]	No	Yes	No	Yes	Yes	3	Average
Metras et al. [38]	Yes	Yes	NA	Yes	Yes	4–5	High
Mekonnen et al. [39]	Yes	Yes	Yes	No	No	3	Average
Wondimu et al. [40]	Yes	Yes	Yes	Yes	No	4	High
Tombari et al. [41]	No	No	No	Yes	No	1	Low
Fasina et al. [42]	Yes	No	No	Yes	Yes	3	Average
Sanni et al. [43]	No	Yes	No	No	No	1	Low
Njoga et al. [44]	Yes	Yes	No	No	Yes	3	Average

Abbreviation: NA, not applicable.

**Table 3:** Farm biosecurity measures studied for a possible association with a disease in livestock in Africa.

No.	Country	Animal species	Study period	Number of farms included	Disease/pathogen studied	Biosecurity or management measures associated with the disease	Reference
1	Nigeria	Pig	Not reported	Not reported	Africa swine fever	Restricted access, fence around premises, gate at the entrance, presence of foot batch/dips, change solution in foot pans regularly, records kept, food and water control, quarantine of newly purchased pigs, final cleaning, routine cleaning, cleaning and disinfection of drinkers and feeders, wash/disinfect equipment and tools, prompt disposal of dead animals, sufficient space for pigs, use of disinfectants, not mixed pigs of different ages, all-in all-out, movement from young to old pigs, change rubber boots, change clothes, separate/isolate sick pigs, consultations and visits of veterinarian/para-veterinarian when animals are sick, downtime of >2 weeks, pests and rodents control, periodical evaluation and audit of biosecurity measures	[42]

2	Uganda	Pig	June to October 2010 and March to June 2011	474	Africa swine fever	Pests present on farm, origin of stock replacement, borrow of boar, wild pig contact	[29]

3	Nigeria	Broiler, layer and turkey	April to May 2019	110	Avian influenza	Egg crate at the farm gate, fence type, sharing of fence with another poultry farm, vehicles at farm gate, poultry kept in closed premises, absence of holes in premises roof/walls, presence of open water reservoir on farm, staff habits—wash hands before handling birds, change shoes before entering poultry premises, use footbath before entering poultry premises, wear gloves when handling birds, visiting hatcheries, closed facilities for carcass disposal	[38]

4	Tunisia	Layer, broiler, broiler breeder and turkey	October 2010 to May 2011	400	Avian influenza	Contact with migratory birds, owners/workers circulating between farms, sanitary and management failures	[41]

5	Egypt	Duck	March to September 2021	20	*Salmonella*, *E. coli*, *Staphylococcus*, *Pasteurella*, avian influenza viruses (H9 and H5) and duck enteritis virus	Good/poor biosecurity	[30]

6	Ethiopia	Dairy cattle	Not reported	174	Bovine tuberculosis	Good/poor biosecurity	[39]

7	Egypt	Cattle	January to December 2017	Not reported	Lumpy skin disease	Cleaning pen periodically, isolation of diseased animals, personal hygiene, carcass disposal	[35]

8	Uganda	Dairy and beef cattle	January to March 2015	144	Brucellosis, salmonellosis and bovine viral diarrhoea	Sharing pasture with other herds	[31]

9	Uganda	Pig	October to December 2019	90	Coinfections of respiratory pathogens and gastrointestinal parasites	Hygiene and drug used	[34]

10	Ethiopia	Broiler	November 2018 to April 2019	6	Coccidiosis	Good/poor management, type of housing system	[40]

11	Egypt	Broiler, layer and duck	January 2017 to August 2018	74	*Eimeria* spp. and *Cryptosporidium* spp.	Restrictions to visitors, multiple species, isolation and quarantine of new birds, fences around premises, use of borrowed equipment/sprayers, cleaning and disinfection of farm equipment, cleaning and disinfection of poultry houses, method of disinfection, presence of foot bath, method of disposal of farm wastes, method of disposal of dead bird, presence of neighbouring farms (within 500 m), trees around the farm, grass around the farm, different age groups, isolation pen for diseased birds, rodent control, bedding material, all in all out system	[33]

12	Nigeria	Broiler, layer and turkey	December 2017 to May 2018	15	Gastrointestinal helminths⁣^a^	Presence of other animals in the farm, housing type, distance to waste area (meters), frequency of pen cleaning	[37]

13	Egypt	Broiler, layer and duck	January 2017 to August 2018	74	Poultry mites	Restrictions to visitors, multiple species, isolation and quarantine of new birds, fence around premises, contact with birds, use of borrowed equipment/sprayers, cleaning and disinfection of farm equipment, cleaning and disinfection of poultry houses, method of disinfection (spraying), presence of foot bath, method of disposal of farm wastes, method of disposal of dead birds, neighbouring farms, different age groups, ponds around the farm, isolation pen for diseased birds, rodent control, farm housing system, bedding material used, all in all out system	[36]

14	Egypt	Broiler	March 2018 to April 2019	9	Salmonellosis	Attraction of wild birds, prevention of wild birds, measures for farmworkers, measures for new poultry on farm, measures for farm visitors	[32]

15	Nigeria	Broiler, layer, others	Not reported	1000	*Salmonella enterica*	Implementation and adherence (partial, no or total) to biosecurity	[43]

16	Nigeria	Broiler and layer	Not reported	60	Thermophilic *Campylobacter*	Sanitization of drinking water, rearing other animals alongside poultry, quarantine of exposed or restocking birds, rearing of birds of different ages, presence of rodents and wild birds in the farm, thinning (partial depopulation of the flock), overstocking/overcrowding	[44]

⁣^a^*Ascaridia galli*, *Heterakis gallinarum*, *Strongyloides avium*, *Raillietina tetragona*, *Subulura brumpti;* good or medium biosecurity/management: implementation of at least half of the biosecurity or management measures evaluated; poor biosecurity/management: implementation of less than half of the biosecurity or management measures evaluated.

**Table 4:** Farm biosecurity measures reported to be significantly associated with livestock diseases in Africa.

Disease	Protective factors		Risk factors	
	**Internal biosecurity**	**External biosecurity**	**Internal biosecurity**	**External biosecurity**
Africa swine fever	- Food and water control - Isolation of sick pigs - Movement from young to old pigs - Not mixing pigs of different ages - Presence of foot bath/dips	- Presence of a gate at the entrance	Downtime of >2 weeks	- Pest and rodent control - Replacement stock (from neighbouring farms)

Avian Influenza	Absence of holes in roof/walls of premises	- Vehicles at farm gate - Traders at farm gate - Change shoes before entering poultry premises - Workers circulating between farms - Absence of holes in roof/walls of premises - Staff's wash hands before handling birds	Poor biosecurity	- Share fence with another poultry farm - Egg crates entering farm - Number of traders visiting per week

Coccidiosis	- Presence of isolation pen - Cleaning and disinfection of farm equipment - Different age groups - Presence of multiple species	- Farm wastes disposal in domestic rubbish - Dead birds throwing in canals - Farm wastes used as land fertilizer - Use of all in all out system - Rodent control - Wood shaving used as bedding material - Presence of grass around the farm	- Good management - No cleaning and disinfection of poultry houses - No use of borrowed sprayers	- No restrictions to visitors - No hay as bedding material - No burning of dead birds - No burying of dead birds - No trees around the farm - No farm wastes in fish farm - No foot baths - Dead birds not used to feed dogs - Medium management

*Note:* Good or medium biosecurity/management: implementation of at least half of the biosecurity or management measures evaluated; poor biosecurity/management: implementation of less than half of the biosecurity or management measures evaluated.

**Table 5:** Results of the meta-analysis of the selected studies and strength of the association between biosecurity measures and diseases in livestock farms in Africa.

Disease	Biosecurity or management measure	Heterogeneity test	OR (95% CI)	*p*-Value
African swine fever	Visitors and farmworkers	*p* < 0.00001, *I*^2^ = 58%	0.74 [0.49, 1.13]	0.74
	Vermin and bird control	*p* < 0.00001, *I*^2^ = 96%	1.55 [0.69, 3.49]	0.29
	Transport of animals, removal of carcasses and manure	*p*=0.52, *I*^2^ = 0%	0.33 [0.12, 0.88]	<0.0001
	Cleaning and disinfection	*p* < 0.00001, *I*^2^ = 94%	0.79 [0.50, 1.24]	0.30
	Measures between compartments, working lines and use of equipment	*p*=0.00001, *I*^2^ = 95%	0.52 [0.38, 0.72]	0.25

Avian influenza	Visitors and farmworkers	*p* < 0.00001, *I*^2^ = 96%	0.48 [0.28, 0.82]	0.007
	Infrastructure and biological vectors	*p* < 0.00001, *I*^2^ = 97%	1.20 [0.68, 2.13]	0.22
	Poor biosecurity^a^	/	1.74 [1.23, 2.48]	0.002

Coccidiosis	Removal of manure and carcasses	*p* < 0.07, *I*^2^ = 46%	0.94 [0.64, 1.36]	0.73
	Cleaning and disinfection	*p*=0.51, *I*^2^ = 0%	1.07 [0.48, 2.38]	0.86
	Infrastructure and biological vectors	*p* < 0.19, *I*^2^ = 29%	1.00 [0.63, 1.58]	0.98
	Medium management^a^	/	4.20 [2.40, 7.40]	0.001
	Good management^a^	/	1.50 [0.90, 2.50]	0.07

^a^Data retrieved from the original paper; good or medium management: implementation of at least half of the management measures evaluated; poor biosecurity: implementation of less than half of the biosecurity measures evaluated; /: no information available in the paper.

## Data Availability

The data that support the findings of this study are available from the corresponding author upon reasonable request.
